# The prevalence and risk factors of sarcopenia among Thai community-dwelling older adults as defined by the Asian Working Group for Sarcopenia (AWGS-2019) criteria: a cross-sectional study

**DOI:** 10.1186/s12877-022-03471-z

**Published:** 2022-10-07

**Authors:** Jiraporn Sri-on, Yupadee Fusakul, Thiti Kredarunsooksree, Thitiwan Paksopis, Rasida Ruangsiri

**Affiliations:** 1 Geriatric Emergency Medicine Unit, The Department of Emergency Medicine, Vajira Hospital, Navamindradhiraj University, Bangkok, Thailand; 2 Department of Rehabilitation, Vajira Hospital, Navamindradhiraj University, Bangkok, Thailand; 3 The Department of Orthopedic, Ratchaphiphat Hospital, Bangkok, Thailand; 4 Thai Health Promotion Organization (ThaiHealth), Bangkok, Thailand

**Keywords:** Sarcopenia, Community-dwelling older adults, Urban, Risk factors

## Abstract

**Background:**

This study aimed to determine the prevalence and risk factors for sarcopenia and severe sarcopenia among urban community-dwelling adults in Thailand, using the Asian Working Group for Sarcopenia (AWGS-2019) criteria.

**Methods:**

This cross-sectional study comprising 892 older adults aged > 60 years analyzed data from a cohort study (Bangkok Falls study; 2019–2021). The appendicular skeletal muscle mass was evaluated using the Bioelectrical Impedance Analysis (BIA) method. Physical performance and muscle strength were evaluated using the five-time sit-to-stand and handgrip strength tests, respectively. Logistic regression was used to determine the factors associated with sarcopenia.

**Results:**

The prevalence rates of sarcopenia and severe sarcopenia were 22.2% and 9.4%, respectively. Age ≥ 70 years (adjusted odds ratio (aOR), 2.40; 95% confidence interval (CI), 1.67–3.45), body mass index (BMI) of < 18.5 kg/m^2^ (aOR, 8.79; 95% CI, 4.44–17.39), Mini Nutritional Assessment (MNA) score of < 24 (aOR, 1.75; 95% CI, 1.24–2.48), and a six-item cognitive screening test score of ≥ 8 (aOR, 1.52; 95% CI, 1.08–12.15) were associated with sarcopenia. Likewise, age ≥ 70 years, BMI < 18.5 kg/m^2^, and an MNA score of < 24 predicted severe sarcopenia.

**Conclusion:**

One-third of the urban community-dwelling older Thai adults had sarcopenia or severe sarcopenia. The age ≥ 70 years, low BMI, and inadequate nutrition increased the risk of both sarcopenia and severe sarcopenia while impaired cognitive functions predicted only sarcopenia in this population.

## Background

Sarcopenia is an age-related progressive disease with loss of skeletal muscle [1, 2]. In 2010, the European Working Group on Sarcopenia in Older People (EWGSOP) developed an algorithm for the diagnosis of sarcopenia, which included the presence of low muscle mass, strength, and physical performance [1]. Since then, the number of studies on sarcopenia has been growing worldwide [3]. The categorization of sarcopenia in the Asian population requires some deliberation due to cultural, lifestyle-related, and anthropometric contrasts with the Western population. In 2014, the Asian Working Group for Sarcopenia (AWGS) proposed an algorithm for the diagnosis of sarcopenia (based on data from the Asian population), which was similar to that proposed by EWGSOP. Recently, in 2019, the AWGS updated the consensus on the diagnosis of sarcopenia and treatment in the Asian population [2].

A systematic review and meta-analysis [4] showed that the pool prevalence of sarcopenia varied from 9.9 to 40.4%, depending on the definition used. A study in Spain reported a prevalence of 63% in long-term care facilities based on the first published EWGSOP criteria [5]. Using the AWGS algorithm, a study from West China found a prevalence of 19.3% among 4,500 community-dwelling older adults aged > 50 years [6]. Recently, a large cohort study conducted among Korean community-dwelling older adults aged ≥ 70 years reported a prevalence of 21.3 and 13.8% in males and females, respectively, based on the AWGS-2019 criteria [7].

Body mass index (BMI) and old age have been indicated as risk factors for sarcopenia [8, 9]. Male gender had been identified as one of the risk factors for sarcopenia with inconsistent results [10, 11], which could be attributed to ethnic differences among the studied populations. Chronic conditions such as congestive heart failure, diabetes, hyperlipidemia, arterial stiffness, malnutrition, and hematological conditions were found to be associated with a decline in muscle mass and sarcopenia [9, 12]. Thailand is a middle-income country with an aging society. The prevalence of sarcopenia among Thai community-dwelling older adults was found to range from 16.1 to 30.6% [13, 14], depending on the definition used in the studies.

The present research aims to analyze a cohort study called the “Bangkok Falls study” [15] using the AWGS-2019 definition and algorithm to determine the prevalence and risk factors of sarcopenia and severe sarcopenia among urban community-dwelling older adults in Thailand.

## Methods

### Study design

The present study was part of the “Bangkok Falls study,” a population-based cohort study that began in 2019–2021 intending to identify and enhance the factors that contribute to falls and aging among community-dwelling older adults aged 60 years and older [15]. The sample in the present study comprising older adults who lived in one of five subdistricts in the Dusit District of Bangkok, Thailand, was able to walk at least 6 m and were expected to live in the community for at least 2 years. The informed consent was obtained from all participants. Older adults who were unable to speak Thai, having a severe cognitive impairment (defined using the six-item cognitive screening test [6-ICT] with a score of > 12 points), taking medications that could affect the body composition (e.g., steroids and diuretics), using electronic devices or metal implants, and were blind or deaf were excluded from the study. The study protocol was approved by the Vajira Hospital Institutional Review Board (IRB) of the university where this study was conducted. The assigned IRB was number 107/2562.

### Data collection at the hospital

Physicians and research assistants (RAs) were trained by a physiotherapist to evaluate the physical performances of the participants before the physical examination. The physical examination was performed by two emergency physicians who were experienced in taking care of older adults for at least 5 years, two RAs with a Bachelor’s degree in health science, and experienced nurse practitioners. The intraclass correlation (ICC) was calculated for the measurement of hand grip strength, and time of the five-time sit-to-stand test.

(The ICC was 0.98 and 0.96 respectively).

### Sarcopenia definition

Sarcopenia was defined according to the definition proposed by the AWGS-2019 [2], which included an age-related decrease in skeletal muscle and muscle strength and/or low physical performance. Sarcopenia was defined as having a low appendicular skeletal muscle mass (ASM) with either low muscle strength or low physical performance. Severe sarcopenia was defined as having a low ASM with both low muscle strength and low physical performance.

The diagnostic criteria for sarcopenia were as follows:

### Screening for cases (possible sarcopenia)

The cases were screened by measuring the calf circumferences of the males (M; < 34 cm) and females (F; < 33 cm).

### Muscle strength measurement

The muscle strength was evaluated using the handgrip strength test. *The* handgrip *strength* was measured by trained RAs using the grip dynamometer model TK-1201 (TAKEI KIKI KOGYO, Japan). First, the pointer was set at 0 kg. The participants were instructed to stand with their backs straight and stretch both arms along with the body and thigh. Then, they were asked to use the dominant hand to lift the handgrip dynamometer and squeeze it for 10–15 s. The measurements were performed twice, and the maximum weight measure from hand grip strength was used (abnormalities were identified as M < 28 kg; F < 18 kg).

### Physical performance measurement

The physical performance was evaluated based on the five-time sit-to-stand test, which measures the time it takes to stand five times from a sitting position without using the arms of a straight-backed armchair. The time was measured from the moment the examiner said “start” while the individual was in the sitting position until the individual was sitting following the fifth stand.

### Measurement of appendicular skeletal muscle mass

The appendicular skeletal muscle mass (ASM) was measured using the Bioelectrical Impedance Analysis (BIA; M, < 7.0 kg/m^2^; F, < 5.7 kg/m^2^) performed via the Inbody Dial device (Korea) with a multi-frequency, tetra-polar electrode. The measurement of BIA was performed at 7.00–9.00 am. to avoid the daily time effect of BIA results.

### Risk factors of sarcopenia and severe sarcopenia

Data on the baseline characteristics, Charlson comorbidity index (CCI), medications used, BMI, Berg Balance Scale (BBS), Mini Nutritional Assessment (MNA), 6-ICT, and frailty phenotype which were defined the results as not frail (no criteria present); pre-frail (one or two criteria present); and frail (three or more criteria present), Barthel activity of daily living (ADL), self-reported “daily number of hours sitting (h/day)”, and fall history within the past 1 year were collected from each participant. RAs check the hospital medical record for hospital visits associated with falls in the past year and asked patients directly for the history of falls in the past year.

The MNA score in older adults can be used to determine the nutritional status (MNA, ≥ 24), malnutrition risk (MNA, 17–23.5), and protein-calorie malnutrition (MNA, < 17).

A medication review was performed during the community and hospital visits. The RAs examined all the containers for the prescriptions, over-the-counter medicines, and herbal medicines used in the previous month. At the hospital, the records of the medications used were rechecked by the PI using the electronic medical record system.

Mobility performance, muscle strength, and musculoskeletal examinations were performed to assess muscle strength and muscle power, particularly in the proximal muscles. The mobility performance assessment during the hospital visit included the time taken to time up and go (TUG) and the 4-m test. The light touch sensation was evaluated using the Semmes–Weinstein monofilament test (size, 5.07; weight, 10 g) in both feet, and the results were classified as sensory deficit and no deficit.

### Statistical analysis

The demographic and clinical findings of the participants were described in this study. Continuous variables were expressed as median (interquartile range (IQR), and the categorical variables were expressed as percentages. Differences in the continuous and categorical variables between the two groups were assessed using the Wilcoxon rank-sum test and Chi-square test/Fisher’s exact test, respectively. Logistic regression was used to determine the factors associated with sarcopenia. Multivariate models were developed by adjusting for covariates with a *P* of < 0.1 in the univariate models with a stepwise backward logistic regression. The goodness of fit for the model was tested using the area under the curve (AUC). The AUC for sarcopenia was 0.72 and the AUC for severe sarcopenia was 0.75. The statistical significance was defined as *P* < 0.05. Stata version 15.1 (Stata Corp., College Station, Texas) was used for the analyses.

## Results

### Prevalence of sarcopenia and severe sarcopenia

**Fig. 1:**
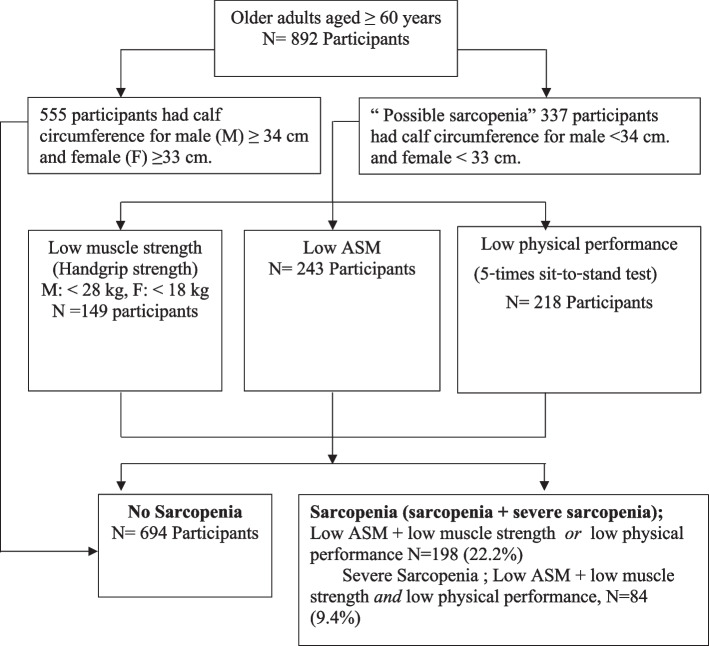
Enrollments of subjects and diagnosis for sarcopenia and severe sarcopenia

A total of 1,001 participants were enrolled and 902 (90.1%) participants completed the one-month follow-up at the hospital. Ten participants were excluded from the study (eight used diuretics and two used steroids). After screening for sarcopenia using the calf circumference measurements, results showed that 337 (37.8%) out of the total 892 participants had a calf circumference of < 34 cm in males and < 33 cm among females. Based on the BIA, handgrip strength, and physical performance evaluations, 198 (22.2%, 95% CI 25.2–31.2) participants had sarcopenia, and 84 (9.4%, 95%CI 7.5–11.5) participants had severe sarcopenia (Fig. 1).

### Baseline characteristics

As shown in Table 1, the average age of the participants was 70 years (IQR 66–75). Those in the sarcopenia group and severe sarcopenia group were older than those in the non-sarcopenia group (median age: sarcopenia 73 (IQR 68–79) years vs. non-sarcopenia 69 (65–74) years; *P* < 0.001, severe sarcopenia 75 (IQR 71–80.5) years vs. non-sarcopenia 69 (65–74) years; *P* < 0.001). The prevalence of sarcopenia among females was 131/614 (21.3%) and among males was 67/278 (24.1%). The prevalence of severe sarcopenia among females and males was 52/614 (8.5%) and 32/278 (11.5%), respectively (Table 2). The median BMI in the sarcopenia group and severe sarcopenia group were less than that in the non-sarcopenia group (sarcopenia, 20.9 (IQR 19.3–22.6) vs. non-sarcopenia 25.5 (23.4–28.7); *P* < 0.001, severe sarcopenia 20.7 (IQR 19.1–22.6) vs. non-sarcopenia 25.5 (23.4–28.7); *P* < 0.001). Those in the sarcopenia groups presented with a 6-ICT of ≥ 8–9 more often than those in the non-sarcopenia group (sarcopenia group, 34.9% vs. non-sarcopenia group, 25.8%; *P* = 0.002). The prevalence of a Charlson co-morbidity score of ≥ 3 was higher in the sarcopenia group compared to that of the non-sarcopenia group (sarcopenia group, 75.3% vs. non-sarcopenia group, 64.4%; *P* = 0.004 and severe sarcopenia group, 85.7% vs. non-sarcopenia group, 64.4%; *P* < 0.001). (Table 1.)

**Table 1:** Baseline characteristic of sarcopenia group and severe sarcopenia group

	Total (*N* = 892)	No Sarcopenia (*N* = 694)	Sarcopenia			*P*-value
			Sarcopenia (*N* = 198)	*P*-value	Severe Sarcopenia (*N* = 84)	
Age (years), median (IQR)	70 (66–75)	69 (65–74)	73 (68–79)	< 0.001	75 (71–80.5)	< 0.001
Female, n(%)	614 (68.8)	483 (69.6)	131 (66.2)	0.35	52 (61.9)	0.15
Education				0.65		0.72
No education/Primary school	574 (64.4)	433 (62.4)	141 (71.2)		57 (67.9)	
Secondary school/diploma	274 (30.7)	224 (32.3)	50 (25.3)		24 (28.6)	
Bachelor degree or higher	44 (4.9)	37 (5.3)	7 (3.5)		3 (3.6)	
Body mass index (BMI), median (IQR)	24.4 (21.9–27.6)	25.5 (23.4–28.6)	20.9 (19.3–22.6)	< 0.001	20.7 (19.1–22.6)	< 0.001
BMI group, n(%)				< 0.001		< 0.001
< 18.5	48 (5.4)	14 (2)	34 (17.2)		18 (21.4)	
18.5–22.9	262 (29.4)	133 (19.2)	129 (65.2)		52 (61.9)	
23–24.9	187 (21)	158 (22.8)	29 (14.7)		12 (14.3)	
≥ 25	395 (44.3)	389 (56.1)	6 (3)		2 (2.4)	
6-CIT score group, n(%)				0.002		0.21
≤ 7	529 (59.3)	433 (62.4)	96 (48.5)		43 (51.2)	
8–9	248 (27.8)	179 (25.8)	69 (34.9)		30 (35.7)	
≥ 10	115 (12.9)	82 (11.8)	33 (16.7)		11 (13.1)	
Activities of Daily Living score < 90	277 (31.1)	209 (30.1)	68 (34.3)	0.26	30 (35.7)	0.33
Charlson Comorbidity Index (CCI) score ≥ 3, n(%)	596 (66.8)	447 (64.4)	149 (75.3)	0.004	72 (85.7)	< 0.001
Frailty phenotype score ≥ 3, n(%)	270 (30.3)	202 (29.1)	68 (34.3)	0.16	34 (40.5)	0.03
Sensory impairment	39 (4.4)	28 (4)	11 (5.6)	0.07	8 (9.5)	0.02
History of falls in the past 1 year	249 (27.9)	197 (27.7)	57 (28.8)	0.76	29 (34.5)	0.16
Had difficulty to exercise	70 (7.9)	56 (7.6)	17 (8.6)	0.66	8 (9.5)	0.55
The daily amount of hours of sitting time (hour/day)				0.03		0.03
< 4	646 (72.4)	514 (74.1)	132 (66.7)		56 (66.7)	
4- < 6	150 (16.8)	114 (16.4)	36 (18.2)		13 (15.5)	
≥ 6	96 (10.8)	66 (10.5)	30(15.1)		15 (17.8)	
Avarage weekly exercise (day/week)				0.23		0.99
No	167 (18.7)	124 (17.9)	43 (21.7)		16 (19.1)	
1–3	162 (18.2)	133 (19.2)	29 (14.7)		15 (17.9)	
4–7	563 (63.1)	437 (63)	126 (63.6)		53 (63.1)	
MNA score				< 0.001		< 0.001
24–30	544 (61)	457 (65.9)	87 (43.9)		32 (38.1)	
17–23.5	333 (37.3)	231 (33.3)	102 (51.5)		49 (58.3)	
< 17	15 (1.7)	6 (0.9)	9 (4.6)		3 (3.6)	
MNA score < 24	348 (39)	237 (34.2)	111 (56.1)	< 0.001	52 (61.9)	< 0.001
Berg Balance Scale (BBS) < 45	118 (13.2)	82 (11.8)	36 (18.2)	< 0.001	27 (32.1)	< 0.001
Time up and go	11.9 (9.9–14.3)	11.6 (9.7–13.9)	12.7 (11–15.9)	< 0.001	13.3 (11.8–17.7)	< 0.001
Proximal muscle weakness	118 (13.2)	85 (12.3)	33 (16.7)	0.11	18 (21.4)	0.02
Polypharmacy ≥ 5	252 (28.3)	203 (29.3)	49 (24.5)	0.21	28 (33.3)	0.28
Herbal used	140 (15.7)	107 (15.4)	33 (16.7)	0.67	10 (11.9)	0.32
Calcium used	103 (11.6)	84 (12.1)	19 (9.6)	0.33	9 (10.7)	0.80

*6-CIT* 6-item Cogitive screening Test, *MNA* Mini Nutritional Assessment

**Table 2:** A comparison prevalence of sarcopenia between male and female

	Male	Female	*P*-value
Bioelectrical impedance analysis (BIA)	*N* = 278 (%)	*N* = 614 (%)	
***Sarcopenia****: Low ASM* + *low muscle strength or* Low physical performance	67 (24.1)	131 (21.3)	0.36
**Severe Sarcopenia:** Low ASM + low muscle strength *and* Low physical performance	32 (11.5)	52 (8.5)	0.15

*ASM* Appendicular skeletal muscle mass

A significantly higher number of participants in the sarcopenia groups and severe sarcopenia group presented with an MNA score of < 24 compared to those in the non-sarcopenia group (sarcopenia group, 56.1% vs. non-sarcopenia group, 34.2%; *P* < 0.001and severe sarcopenia group, 61.9% vs. non-sarcopenia group, 34.2%; *P* < 0.001). The sarcopenia groups and severe sarcopenia group had a higher prevalence of proximal muscle weakness than the non-sarcopenia group (sarcopenia group, 18.2% and severe sarcopenia group, 32.1% vs. non-sarcopenia group, 11.8%; *P* < 0.001and *P* < 0.001, respectively). (Table 1.)

### Risk factors for sarcopenia (*N* = 198) and severe sarcopenia (*N* = 84*)*

The results of the multivariate analysis showed that age [≥ 70 years; adjusted odds ratio (aOR), 2.40; 95% confidence interval (CI), 1.67–3.45], a BMI of < 18.5 (aOR, 8.79; 95%CI, 4.44–17.39), an MNA score of < 24 (aOR, 1.72; 95%CI, 1.21–2.44), and a 6-CIT score of ≥ 8 (aOR, 1.52; 95% CI, 1.08–12.15) were associated with sarcopenia (Table 3). Age (≥ 70 years; aOR, 4.47; 95%CI, 2.47–8.09), BMI of < 18.5 (aOR, 5.77; 95%CI, 2.87–11.56), and an MNA score of < 24 (aOR, 2.02; 95%CI, 1.23–3.30) predicted the incidence of severe sarcopenia (Table 4).

**Table 3:** Univariate and multivariate analysis for risk factors of sarcopenia

	Univariate		Multivariate	
	OR (95%CI)	*P*-value	aOR (95%CI)	*P*-value
Age ≥ 70 vs < 70 year	2.54 (1.81–3.57)	< 0.001	2.40 (1.67–3.45)	< 0.001
Female vs male	0.85 (0.61–1.2)	0.36		
Education				
No education/Primary	1.72 (0.75–3.95)	0.20		
Secondary/diploma	1.18 (0.5–2.8)	0.71		
Bachelor degree or higher	Ref			
BMI: < 18.5 vs ≥ 18.5	10.07 (5.28–19.2)	< 0.001	8.79 (4.44–17.39)	< 0.001
6-CIT score: ≥ 8 vs < 8	1.76 (1.28–2.42)	< 0.001	1.52 (1.08–12.15)	0.02
Charlson Comorbidity Index ≥ 3	1.68 (1.17–2.4)	0.01		
Frailty phenotype score > 3	1.27 (0.91–1.78)	0.16		
Activities of Daily Living score < 90	1.21 (0.87–1.7)	0.26		
MNA < 24	2.46 (1.78–3.39)	< 0.001	1.75 (1.24–2.48)	0.002
Sensory impairment	1.4 (0.68–2.86)	0.36		
Proximal muscle weakness	1.43 (0.93–2.22)	0.11		
Polypharmacy ≥ 5	0.79 (0.55–1.14)	0.01		
Herbal used	1.1 (0.72–1.68)	0.67		
Calcium used	0.77 (0.46–1.3)	0.33		
Had difficulty to exercise	1.14 (0.64–2.01)	0.66		
The daily amount of hours of sitting time ≥ 6 h	1.7 (1.07–2.7)	0.03		
Avarage weekly exercise (day)				
No	Ref			
1–3	0.63 (0.37–1.07)	0.09		
4–7	0.83 (0.56–1.24)	0.37		
History of falls in the past 1 year	1.06 (0.75–1.5)	0.76		

Multivariate models were developed by adjusting for covariates with *p* < 0.1 in univariate models with stepwise backward LR

*BMI* Body mass index, *6-CIT* 6-item Cogitive Impairment Test, *MNA* Mini Nutritional Assessment, *OR* Odds ratio, *aOR* Adjusted odds ratio,*95%CI* 95% confidence interval

**Table 4:** Univariate and multivariate analysis for risk factors of severe sarcopenia

	Univariate		Multivariate	
	OR (95%CI)	*P*-value	aOR (95%CI)	*P*-value
Age ≥ 70 vs < 70 year	4.49 (2.52–7.98)	< 0.001	4.47 (2.47–8.09)	< 0.001
Female vs male	0.71 (0.45–1.13)	0.15		
Education				
• No education/Primary	1.51 (0.45–5.02)	0.50		
• Secondary/diploma	1.31 (0.38–4.56)	0.67		
• Bachelor degree or higher	Ref			
BMI: < 18.5 vs ≥ 18.5	7.07 (3.74–13.36)	< 0.001	5.77 (2.87–11.56)	< 0.001
6-CIT score: ≥ 7 vs < 7	1.44 (0.92–2.26)	0.11		
Charlson Comorbidity Index ≥ 3	3.25 (1.74–6.09)	< 0.001		
Frailty phenotype score > 3	1.65 (1.04–2.61)	0.03		
Activities of Daily Living score < 90	1.26 (0.79–2.02)	0.33		
MNA score < 24	2.81 (1.77–4.47)	< 0.001	2.02 (1.23–3.30)	0.005
Sensory impairment	2.63 (1.17–5.94)	0.02		
Proximal muscle weakness	1.93 (1.1–3.39)	0.02		
History of falls in the past 1 year	1.41 (0.88–2.27)	0.16		
Polypharmacy ≥ 5	1.30 (0.81–2.10)	0.28		
Herbal used	0.70 (0.35–1.4)	0.32		
Calcium used	0.91 (0.44–1.88)	0.80		
Had difficulty to exercise	1.27 (0.58–2.74)	0.55		
The daily amount of hours of sitting time ≥ 6 h	1.95 (1.07–3.57)	0.03		
Avarage weekly exercise (day)				
• No	Ref			
• 1–3	0.96 (0.46–2.02)	0.92		
• 4–7	0.98 (0.54–1.77)	0.95		

Multivariate models were developed by adjusting for covariates with *p* < 0.1 in univariate models with stepwise backward LR

*BMI* Body mass index, *6-CIT* 6-item Cogitive Impairment Test, *MNA* Mini Nutritional Assessment, *OR* Odds ratio, *aOR* Adjusted odds ratio,*95%CI* 95% confidence interval

## Discussion

The main objective of this study was to determine the prevalence of sarcopenia and severe sarcopenia among community-dwelling older adults in a Thai urban area using the AWGS-2019 definition. In addition, correlations between sarcopenia, severe sarcopenia and age, nutritional status, cognitive function, and physical activity were evaluated.

### Prevalence of sarcopenia and severe sarcopenia

The prevalence of sarcopenia as defined by AWGS-2019, among urban Thai community-dwelling older adults was 22.2% (males, 24.1%; females, 21.3%). The prevalence of sarcopenia in this study was not different from that reported in another study comprising community-dwelling adults aged ≥ 70 years in Korea (males, 20.1%; females, 29.2%) [7]. The prevalence of sarcopenia and severe sarcopenia in the current study was higher than that reported among ≥ 60-year-olds in the study by Wu X et al. (sarcopenia, 18.6% and severe sarcopenia, 8%) [16]. The authors used the AWGS-2019 criteria for the diagnosis of sarcopenia. However, a validated anthropometric equation was used in the Chinese population to estimate the ASM instead of dual X-ray absorptiometry or bioelectrical impedance as per the AWGS-2019 recommendations. The prevalence of sarcopenia in this study was higher than that reported by Therakomen V, et al. in a Thai community-dwelling outpatient older adults; they found that the prevalence of sarcopenia according to the AWGS-2019 was 8.8% and severe sarcopenia was 1.2% [17]. This discrepancy in the prevalence might be attributed to the difference in the age of the participants between the two studies (median age in the current study, 70 years; mean age in the Therakomen et al. study, 66.89 years). In addition, Therakomen V, et al. study evaluated only primary sarcopenia, the study excluded chronic diseases such as chronic obstructive pulmonary disease (COPD), stroke, parkinsonism, and autoimmune diseases.

### Sarcopenia and severe sarcopenia risk factors

Advanced age was associated with both sarcopenia and severe sarcopenia in this study, which was similar to that reported by Wu et al. [16]. The results of the present study are consistent with those of a meta-analysis of 34 studies, which found that age was associated with the incidence of sarcopenia among community-dwelling older adults (OR, 1.12.; 95% CI, 2.55–5.60) [18]. The previously published data evaluated the overall sarcopenia, there was no separation between the risk factors of sarcopenia and severe sarcopenia.

An MNA score of < 24 (at risk of malnutrition) was independently associated with sarcopenia (aOR, 1.72; 95% CI, 1.21–2.44) and severe sarcopenia (aOR, 1.98 (95% CI, 1.20–3.25) in this study. These results were consistent with those reported by Gao et al., who reported that malnutrition or at risk of malnutrition (defined using MNA-SF) was associated with sarcopenia (OR, 3.53; 95% CI, 1.68–7.41) in urban and rural Chinese older adults [19]. Sousa–Santos et al. demonstrated the association between malnutrition or at risk of malnutrition (determined using MNA-SF) and sarcopenia (OR, 1.86; 95% CI, 1.01–3.43) among Portuguese older adults [20]. Likewise, Kurose et al. found that malnutrition (defined using a total cholesterol level of < 150 mg/dl and/or albumin level of < 3.5 g/dl) was associated with sarcopenia (aOR, 2.42; 95% CI, 1.04–5.60) among community-dwelling older adults in Japan [21]. Furthermore, a meta-analysis of 10 studies showed that malnutrition/malnutrition risk increased the risk of sarcopenia (OR, 2.99; 95%CI, 2.40–3.72) among community-dwelling older adults [18].

In the present study, a low BMI (< 18.5 kg/m^2^) increased the risk of developing sarcopenia. Similar findings have been reported among Japanese community-dwelling older adults [22, 23], Iranian older adults [24], and Italian community-dwelling older adults [25]. A Chinese study [26] reported that a high BMI was a risk factor for slow gait speed, whereas a high BMI acted as a protective factor for the loss of skeletal muscle mass.

Cognitive impairment (6-CIT score, ≥ 8) was associated with sarcopenia; an association with severe sarcopenia was observed in the univariate but not multivariate analysis. Probably due to the lower rate of severe sarcopenia. Sarcopenia was found to be significantly related to cognitive decline in a Thai local community [13], community-dwelling Japanese older adults [27], Taiwanese community-dwelling older adults [28, 29], and Korean older women [30]. A pool systematic review of six studies showed that cognitive impairment was significantly associated with sarcopenia (OR, 1.62; 95% CI, 1.05–2.51) [18].

Sarcopenia has a poor prognosis [31], future studies should be emphasizing the role of tailored risk factors screening, including malnutrition and dysphagia [32, 33], in older adults in order to perform a personalized approach including physical exercise and nutritional intervention [34].

The strength of this study is that it was conducted using a large sample size of urban older adults. However, this study had some limitations. First, only ambulatory community-dwelling Thai older adults were included in the study; hence, the results may not be generalized. The prevalence of sarcopenia in this study was probably less than the actual prevalence because it excluded older adults with severe cognitive impairment who could not perform any physical functions, which might represent selection bias in this study. We could not be evaluated the actual volume status by the time that the RAs performed the BIA measurement. The results may have an effect on the difference in volume status. The original cohort “Bangkok fall study” [15] evaluated volume status and intracellular dehydration using serum osmolarity. The results came back 2–3 h after the blood draw. The actual times for physical activities relied upon the memories of the participants and not the exact scheduled time. The cross-sectional design has a limitation in elucidating the causal relationship between risk factors and sarcopenia. This study did not evaluate the levels of inflammatory cytokines, which could contribute to the development of sarcopenia.

## Conclusions

The prevalence of sarcopenia and severe sarcopenia among Thai urban community-dwelling older adults using the AWGS-2019 definition were 22.2 and 9.4% respectively. The risk of sarcopenia and severe sarcopenia increased among those with age ≥ 70 years, low BMI, and inadequate nutrition. While impaired cognitive function increased the risk only for sarcopenia. These findings might prove beneficial for the early identification of individuals at risk of sarcopenia and severe sarcopenia and aid in the implementation of interventions to prevent this disease among urban older adults.

## Data Availability

All data generated and/or analysed during the current study are not publicly available due to the plan of analysis in others manuscripts but are available from corresponding author on reasonable request.

## Abbreviations

AWGS-2019: The Asian Working Group for Sarcopenia criteria
BIA: Bioelectrical Impedance Analysis
aOR: Adjusted odds ratio
BMI: Body mass index
MNA: Mini Nutritional Assessment score
6-ICT: A Six-item cognitive screening test
EWGSOP: The European Working Group on Sarcopenia in Older People
IRB: Institutional Review Board
RAs: Research assistants
ICC: The intraclass correlation
ASM: Appendicular skeletal muscle mass
CCI: Charlson comorbidity index
BBS: Berg Balance Scale
ADL: Activity of daily living
TUG: Time up and go
AUC: Area under the curve
